# Superior Mesenteric Artery (SMA) Syndrome With Enterocutaneous Fistula in a Young Woman: A Rare Association

**DOI:** 10.7759/cureus.39696

**Published:** 2023-05-30

**Authors:** Shreyas N, Atul Jhanwar, Narender Singh

**Affiliations:** 1 Surgery, Ananta Institute Of Medical Sciences and Research Centre, Udaipur, IND; 2 General Surgery, Dr. Sampurnanand Medical College, Jodhpur, IND

**Keywords:** wilkie's syndrome, multidisciplinary approach, surgical procedures, enterocutaneous fistulas, superior mesenteric artery syndrome

## Abstract

Superior mesenteric artery (SMA) syndrome is also known as Wilkie's syndrome. Sometimes it is the cause of obstruction in the duodenum. In SMA syndrome, the acute angulation of the SMA against the abdominal aorta can prevent duodenal contents from draining into the jejunum (upper small intestine); hence inadequate intake of nutrition leads to weight loss and malnutrition. This is primarily attributed to the loss of intervening pad of mesenteric fat tissue due to various debilitating conditions.

Abnormal connections between the intra-abdominal gastrointestinal tracts and skin over the abdomen are known as enterocutaneous fistulas (ECF).

In this case report, a 37-year-old woman with a history of chronic dull pain in the upper abdominal region over the last seven months, who also complained of bloating, infrequent vomiting, nausea, and upper abdominal fullness for the same amount of time, was seen in the emergency room. Her symptoms had deteriorated by the time she approached the hospital. Additionally, she reports having had a foul-smelling, purulent discharge for the past five years right below the umbilicus. Upon close investigation, it was determined to be feces, and it was later discovered to be a low-output enterocutaneous fistula. She describes having an exploratory laparotomy and adhesiolysis for an intra-abdominal abscess and an acute intestinal obstruction caused by adhesions. This case emphasizes the provocation given a diagnosis of SMA syndrome with enterocutaneous fistula and demands increased awareness of this entity. This will ameliorate early identification to reduce immaterial tests and irrelevant treatments.

## Introduction

The superior mesenteric artery (SMA) syndrome is vascular compression of the third part of the duodenum in the angle between the abdominal aorta and the superior mesenteric artery. It was first described by Carl Freiherr Von Rokitansky in 1842 and first published by Dr. Wilkie, so it is also called Wilkie's Syndrome. It is also called arterio-mesenteric duodenum compression syndrome, chronic duodenal ileus, and cast syndrome. It is a very rare syndrome with a prevalence of 0.0024-0.3% [1-2]. The majority of SMA cases are mostly seen in women who lost their weight voluntarily or secondary to other medical conditions like anorexia, malignancy, malabsorption, and gastrectomy [3]. In all of the above cases, there will be a loss of perivascular mesenteric fat, which leads to a reduction of the aortic mesenteric angle (normal angle 25 degrees to 45 degrees). When it reduces below 20 degrees, it's called SMA syndrome [4]. Due to duodenal compression, intestinal obstructive symptoms occur. Patients usually present with chronic epigastric pain, bloating, vomiting, and rapid weight loss [5]. In SMA syndrome, sometimes, left renal vein compression occurs, which causes microhematuria, varicocele, left flank pain, and vascular thrombosis [6]. 

Initially, non-surgical treatment like frequent small feedings with supplemental high-calorie diet or high-calorie intravenous feeding (TPN) followed by postural therapy is effective. In some cases, surgical interventions like duodenojejunostomy or Roux-en-Y reconstruction may be required. Endovascular stent placement is one of the new modes of treatment these days [7]. Adequate nutrition and postural measures can lead to weight gain and can resolve symptoms. An aberrant connection between the gastrointestinal system and the skin of the abdomen is known as an enterocutaneous fistula. It usually occurs as a complication of previous abdominal surgery like laparotomy. Usually, one-third of fistulas close spontaneously. The fistulas may be associated with chron disease, diverticular diseases, and radiation enteritis [8]. Enterocutaneous fistulas can be caused by nutritional deficiency, septic problems, and various other factors. This fistula has to be repaired to facilitate enteral nutrition and restore digestive tract integrity. A multi-specialty team is required to treat an enterocutaneous fistula.

## Case presentation

**Figure 1:**
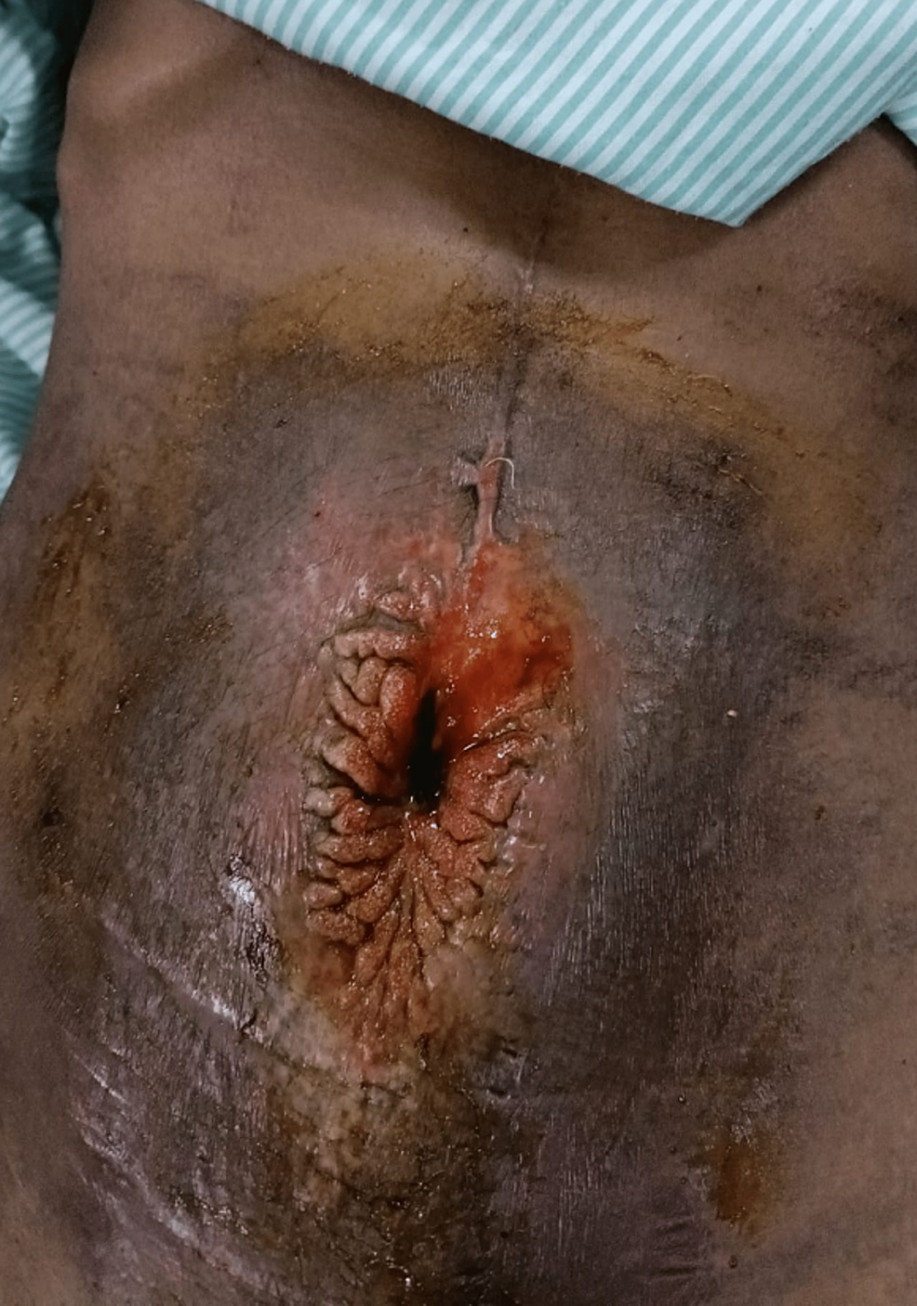
Grossly indurated surrounding skin with erythema in the center and blackish discoloration in the periphery. The center part of the abdomen shows a crater-like depression, which was expressing fecal matter with a foul smell.

A 37-year-old woman arrived at the emergency room complaining of upper abdomen fullness, nausea, occasional vomiting, and a constant dull ache in the epigastric region that had continued for the previous seven months. She also gave a history of gradual loss of weight of around 4 kg over the last five months. She also presents with a history of foul-smelling, purulent discharge just below the umbilicus from five years which was noticed to be fecal matter on careful examination and turned out to be a low-output enterocutaneous fistula. She gave a history of undergoing exploratory laparotomy with adhesiolysis done for acute intestinal obstruction due to adhesions with intra-abdominal abscess five years and three months back. Her prior surgical records indicate that the laparotomy went smoothly and without any intestinal injuries. Since she never saw a doctor again for pus leakage from her abdominal wall following that surgery, the cause of the enterocutaneus fistula remains unknown. She had a history of taking pantoprazole tablets regularly. She was admitted to our surgery department in our institution. As per the clinical examination, her vitals were normal, but her blood pressure was 90/60 mm of hg. Her build was thin, and she was anemic. Her BMI was 18.1kg/m2, and her abdomen was diffusely distended, bilateral flanks were full. Mild guarding and rigidity were present in the upper abdomen. Although there was no peritonitis, the patient had signs of intestinal obstruction. The surrounding skin was grossly indurated with erythema in the center and blackish discoloration in the periphery. The center part of the abdomen shows a crater-like depression, which was expressing fecal matter with a foul smell (Figure 1).

At laboratory evaluation at the time of admission, all parameters were normal except bicarbonate level elevation ( 31 mmol/l) consistent with emesis alkalosis. Her Hb - 9.3 g/dl, WBC -7500/mm3, Na -134 mmol/l, K+ - 3.6 mmol/l. Her liver and renal profile were normal [ Table 1]. 

**Table 1:** Blood investigations of the patient SGOT - serum glutamic-oxaloacetic transaminase, SGPT - serum glutamic pyruvic transaminase, ALP - alkaline phosphatase, WBC - white blood cell, RBC - red blood cell, HB - hemoglobin, MCV - mean corpuscular volume, MCH - mean corpuscular hemoglobin, MCHC - mean corpuscular hemoglobin concentration

Biochemistry and hematology analyses	Results
Renal function test	
Serum urea	10.89 mg/dl
Serum creatine	0.54 mg/dl
Serum uric acid	1.33 mg/dl
Electrolytes	
Sodium	134 mmol/L
Potassium	3.6 mmol/L
Chloride	109 mmol/L
Liver function test	
Bilirubin total	0.65 mg/dl
Bilirubin direct	0.08 mg/dl
Bilirubin indirect	0.06 mg/dl
SGOT	32.13 U/L
SGPT	21.73 U/L
ALP	284.29 U/L
Hematological analyses	
WBC	7.5 x 10^3 cells/cumm
RBC	4.1 millions/cumm
HB	9.3 g/dl
MCV	84.2 fL
MCH	27.2 pg
MCHC	32.3 gm/dl
Platelet count	311.0 x 10^3 cells/cumm
Neutrophils	84.40%
Lymphocytes	11.40%

**Figure 2:**
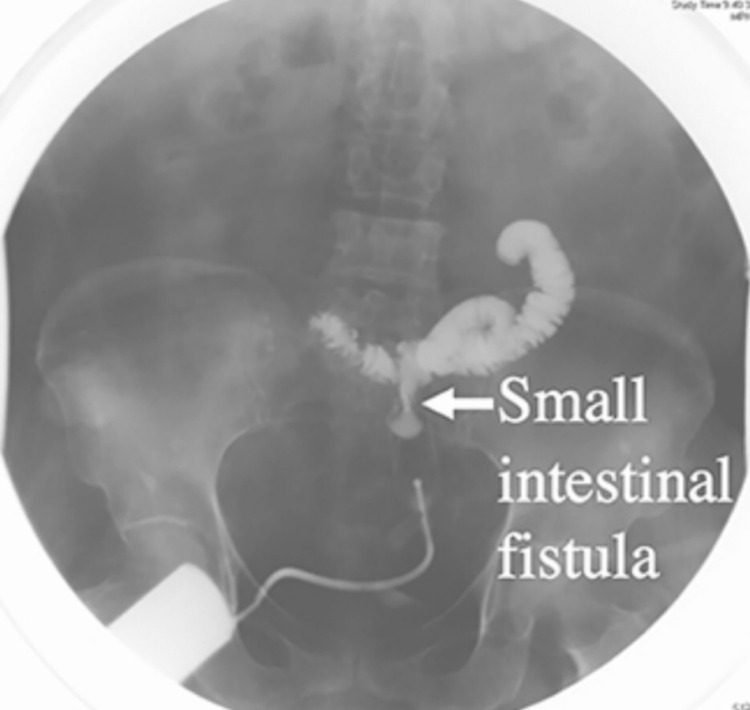
A fistulogram with water-soluble contrast material showing the anatomical pathway of the fistula tract

A fistulogram using water-soluble contrast material showed the anatomical pathway of the fistula tract (Figure 2).

**Figure 3:**
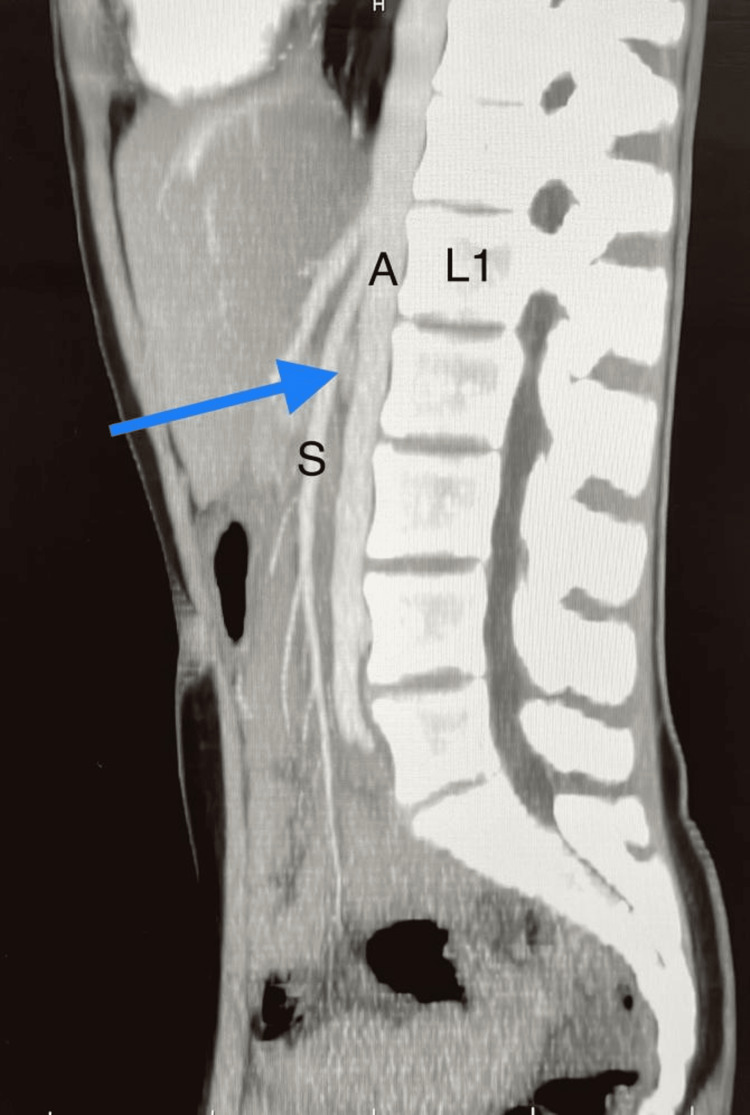
Sagittal CT scan illustrating the duodenum's entrapment at an aorta-SMA angle of 12.9° (2D) at a distance of 4.5 mm SMA - superior mesenteric artery

An acute angle of 12.9 degrees between the superior mesenteric artery and the abdominal aorta was identified on a contrast-enhanced computed tomography (CECT) of the pelvis and abdominal area, along with a depression in the anterior region of the duodenum. The SMA and aorta distance was 4.5 mm, and there was distension of the stomach and proximal section of the duodenum (Figure 3).

**Figure 4:**
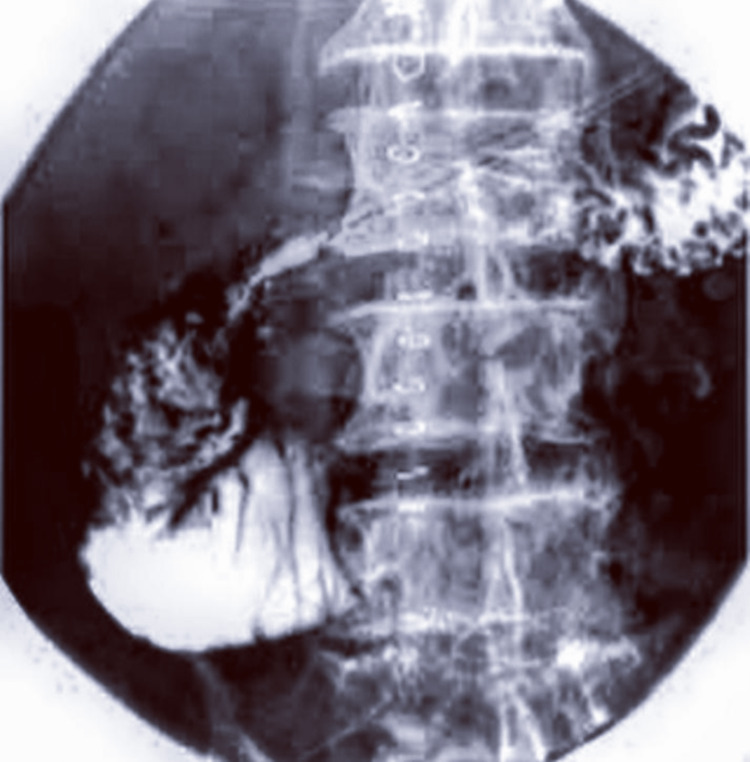
Barium study showing dilated proximal duodenum with narrowing of the third part of the duodenum

So the above clinical features and imaging features were diagnosed as superior mesenteric artery syndrome, and thereafter, delayed transit through a third part of the duodenum was shown in the barium study (Figure 4).

**Figure 5:**
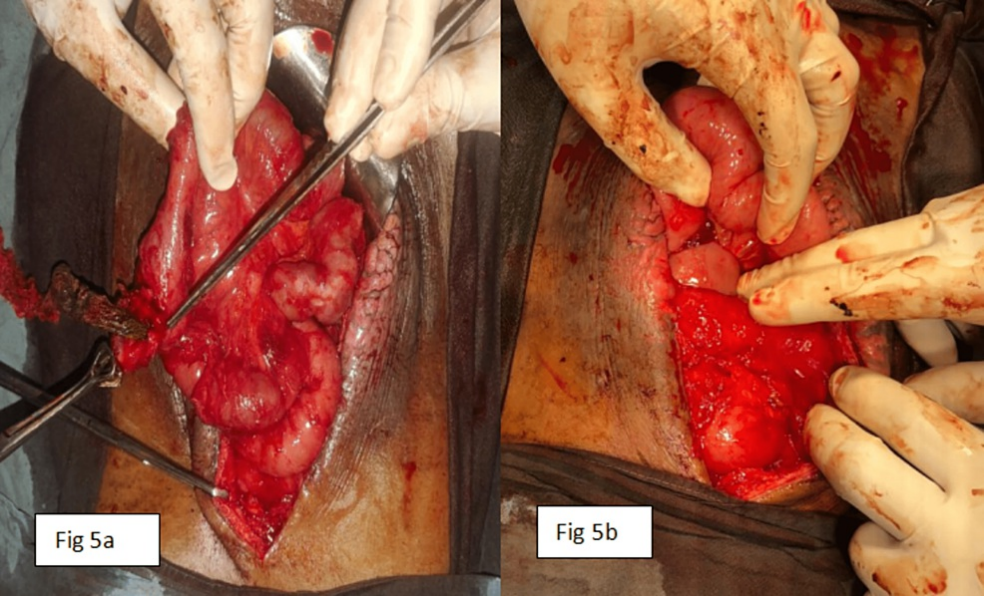
A) ileal segment with fistula opening; B) dilated first and second part of duodenum up to the crossing of the superior mesenteric artery

Initially, the patient was in NPO, and nasogastric suction started to decrease the output. Then, IV fluid, H2 receptor antagonist, and proton pump inhibitors (PPI) were used to reduce fistula output. Later on, surgical intervention was done. An intraoperative ileal segment with a fistulous opening, as well as dilated first and second parts of the duodenum up to the crossing of SMA, was found (Figure 5).

**Figure 6:**
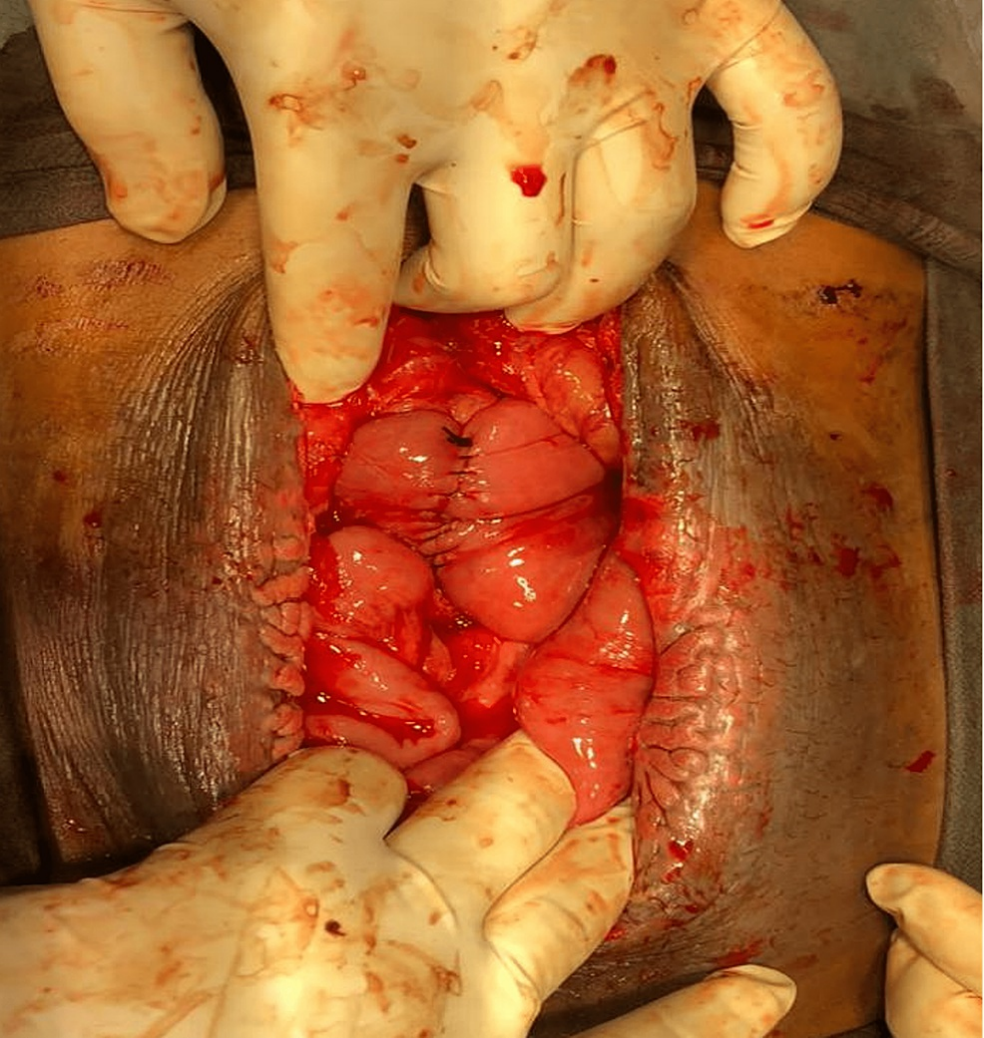
Side-to-side duodenojejunostomy without duodenal mobilization

Then, the patient underwent wedge resection and primary repair of the ileal segment with excision of the fistula and the surrounding skin followed by side-to-side duodenojejunostomy without duodenal mobilization (Figure 6).

**Figure 7:**
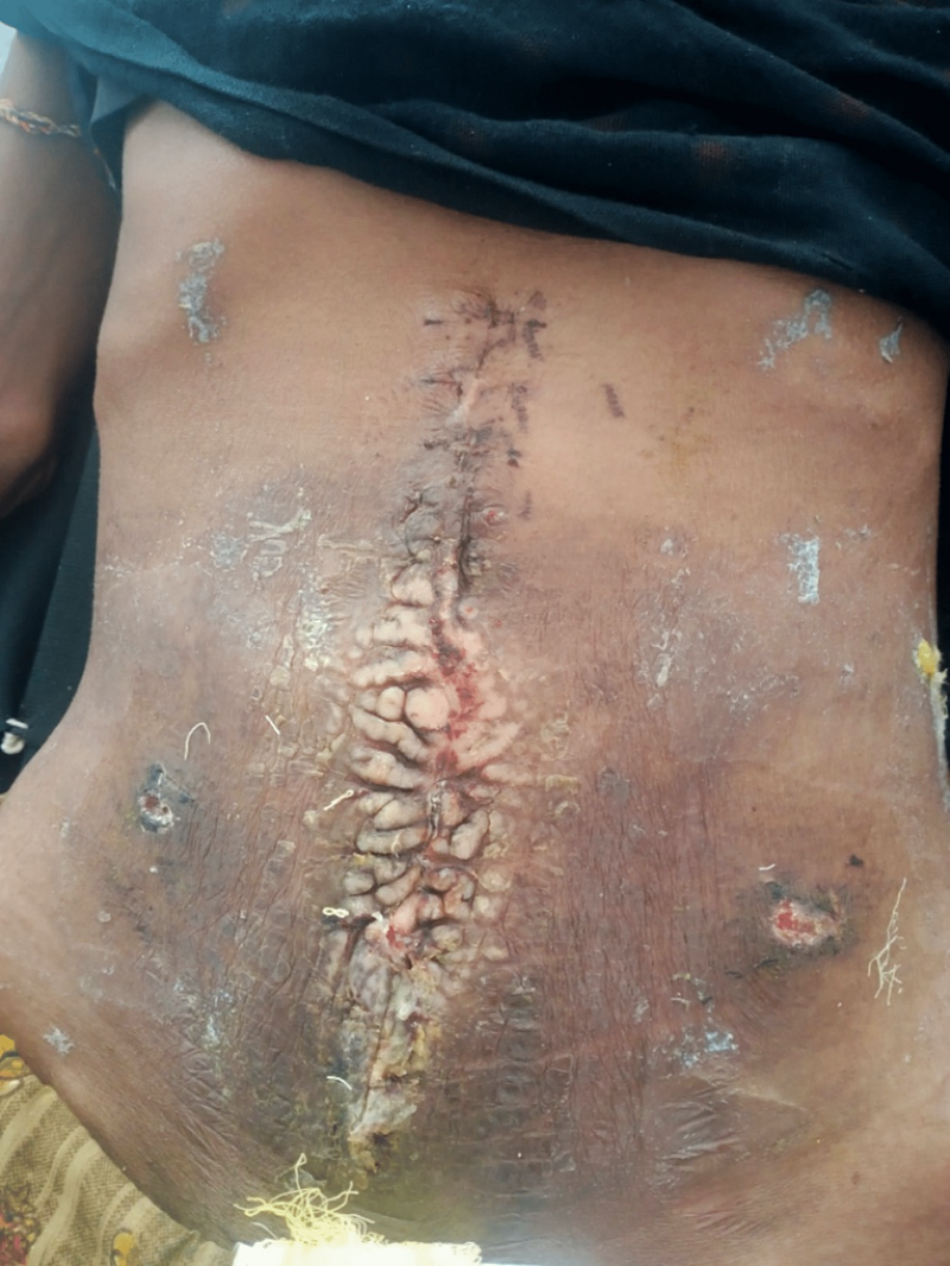
Remodeling of the postoperative wound with freshly healed epidermis to restore the skin's integrity

She was given IV TPN postoperatively. She was also treated with IV antibiotics, IV analgesis, and IV PPI postoperatively. Gradually, she was started on a normal diet, which relieved her symptoms. The recovery time went without a hitch, and she went home on the 10th postoperative day without any discharge from the wound (Figure 7). 

## Discussion

The occurrence rate of superior mesenteric artery syndrome is around 0.003 and 0.3% [9]. The third portion of the duodenum gets trapped between the SMA and the aorta, resulting in obstruction of the upper part of the gastrointestinal tract [2]. In healthy individuals, the superior mesenteric artery and aorta's angle ranges from 38 to 65 degrees; however, in those with SMA syndrome, it is only 7 to 22 degrees. This angle corresponds with the body mass index [10]. The aortomesenteric distance is normally 10 to 28 mm, but with SMA syndrome, it is reduced to 2 to 8 mm [11]. The loss of the mesenteric fat pad around the SMA and behind the abdominal aorta causes the aorta-SMA angle to shrink, which is the primary anatomical feature of SMA syndrome [12].

Congenital or acquired anatomic abnormalities can cause SMA syndrome. Major reductions in weight, nutritional issues, and diseases that lead to cachexia are all linked to SMA syndrome. Treitz ligament shortness or hypertrophy are examples of congenital causes. Scoliosis surgery, peritoneal adhesions, duodenal malrotation, Ladd's bands, and mesenteric root tumour are additional risk factors. Acute presentation of this syndrome is usually characterized by severe intestinal obstruction leading to life-threatening dilatation of the stomach. Chronic cases may present with long-standing vague abdominal symptoms or recurrent episodes of abdominal pain, associated with vomiting, like in our case. The diagnosis of the SMA syndrome is difficult to make clinically as signs and symptoms are vague and non-specific and often delayed due to its insidious presentation. Diagnosis is based on interpreting clinical evidence alongside radiological findings. Diagnosis of SMA syndrome can be done by contrast-enhanced computed tomography scan or magnetic resonance angiography, which shows a constriction at the third section of the duodenum compressed by SMA, with proximal dilatation of the duodenum to compression and permits measurement of the aortomesenteric angle and distance. Barium and endoscopy studies can be used but are often non-specific. Endoscopic examination may visualize complications of the disease like gastric stasis, and biliary reflux [13]. Conventionally, treatment has consisted of conservative measures such as gastric decompression, parenteral nutrition and/or post-pyloric feeding if feasible, followed by oral diet as tolerated [9]. Posturing movements during meals and motility agents may be obliged in some patients. Surgery treatment may be scrutinized given the failure of conservative treatment [14]. In this patient, ileal resection and nasojejunal feeding may be options; however, because of the patient's chronic vomiting and inflated stomach from duodenal compression, duodenojejunal anastomosis may be preferred instead of ileal resection. Duodenojejunostomy is the safe and effective surgical operation of choice to achieve satisfactory decompression of the third part of the duodenum and a functional bypass with a success rate of up to 90% [14]. Strong's method, another minimally invasive surgical alternative, entails lysis of the Treitz ligament with the rallying of the duodenum, although this surgery had a 25% failure rate [2]. Gastrojejunostomy has been halted because it raised the risk of blind loop syndrome and recurrence brought on by the duodenum's restfulness and as a result of the aortomesenteric distance dropping to 10 mm in the postoperative period [2]. 

In this patient, SMA syndrome could have been avoided if weight loss and the disappearance of the mesenteric fat pad had not occurred. For this, prompt detection and treatment of the enterocutanous fistula is crucial, coupled with proper nutritional assistance following a prior laparotomy. 

An enterocutaneous fistula is established when there is a non-typical communication between the gastrointestinal tract and the skin and wound or vice versa. Iatrogenic, or unintentionally occurring during a visceral operation, is the most frequent cause of ECF. One-fourth of these cases may occur spontaneously in most commonly diagnosed inflammatory bowel diseases like Crohn's disease [15]. Most ECFs occur after bowel surgery. Other surgical causes include anastomotic failure, Abdominal wall dehiscence, mesh rupture, and drain puncture. An ECF may also develop from abdominal penetrating trauma. The external cutaneous orifice (EFO), which is located at the anterior or lateral abdomen wall and is surrounded by an inflammatory, sensitive cutaneous area, is the clinical signature. The EFO may occasionally be concealed behind a skin fold or be located at the spot of a recent or well-established prior incision from surgery. Discharge, which can occasionally be purulent in nature or fecal, may be present intermittently or continuously. A high-output fistula has an output of more than 500 mL per 24 hours, whereas a low-output fistula has an output of less than 200 mL per 24 hours. 

The Sepsis, Nutrition, Anatomy, Procedure (SNAP) guideline is used to treat ECF patients since it is successful in doing so. Reanimation is the initial stage, which is subsequently followed by sepsis diagnosis. Imaging to outline the anatomy is done after that if the patient is well-numerified and the effluent from the fistula is well-controlled. Surgery to close the fistula and address the root causes is the last step. Our situation was handled by the aforementioned recommendations, and the prognosis indicated that she would recover without any major complications.

A fistulogram utilizing water-soluble contrast substance is the industry-standard examination for determining the anatomy of a fistula. A fistulogram provides information on the following: 1) the origin of the fistula; 2) the physical characteristics of the fistula, including its measurement, course, and connection to the gastrointestinal; 3) the presence or absence of intestinal integrity; 4) the presence or absence of distal obstruction; 5) absence or presence of inflammation and stricture; and 6) other studies such as abdominal CT scans and MRI of the abdomen also provide convenient information in selected cases [16].

Homeostasis of the whole gastrointestinal tract, resection of the fistula with end-to-end bowel anastomosis, and abdominal wall closure are the three objectives of surgery for an ECF. By using guided probes or methylene blue dye, the fistula tract can be identified. Resection of the fistula followed by end-to-end anastomosis is the preferred approach rather than over-sewing the fistula because recurrent fistula is more likely to occur following over-sewing (36%) than resection (16%) [17]. When trying to close a fistula, abdominal closure is strictly enforced. Skin closure is made more challenging by skin imperfections and the increased possibility of wound infection. Intraoperative problems would manifest as new or recurrent EC fistulas. It would be necessary to have adequate parenteral or enteral nutrition together with a supplement rich in trace minerals and vitamins for the healing of postoperative wounds to be secured in abdominal surgery.

## Conclusions

High clinical intuition is crucial, especially in individuals who have significant weight loss and have signs of stomach distension. Nearly all of the patients who had ECF are underprivileged, lack information, and are neglected, which leads to delayed presentation.

The most favorable diagnostic and treatment outcomes in this frequently misdiagnosed condition come through multifaceted collaboration. Treatment of ECF remains a surgical summons despite the latest improvement in supportive patient care. Despite the most recent advancement in supportive patient care, ECF treatment still necessitates surgery. Planning, thorough dissection, resection of the fistula, anastomosis, and restoration of the abdominal wall are crucial if surgery is necessary. This study emphasizes how challenging it may be to correctly diagnose SMA conditions in a non-traditional clinical context. The purpose of this paper is to raise awareness of a rare condition called duodenal obstruction in order to facilitate early detection and reduce complications.
